# Assessing the Response Results of an mHealth-Based Patient Experience Survey Among People Receiving HIV Care in Lusaka, Zambia: Cohort Study

**DOI:** 10.2196/54304

**Published:** 2024-09-30

**Authors:** Jacob Mutale, Kombatende Sikombe, Boroma Mwale, Mwansa Lumpa, Sandra Simbeza, Chama Bukankala, Njekwa Mukamba, Aaloke Mody, Laura K Beres, Charles B Holmes, Carolyn Bolton Moore, Elvin H Geng, Izukanji Sikazwe, Jake M Pry

**Affiliations:** 1 Data Unit Centre for Infectious Disease Research Lusaka Zambia; 2 Implementation Science Unit Centre for Infectious Disease Research Lusaka Zambia; 3 Analysis Unit Centre for Infectious Disease Research Lusaka Zambia; 4 Social Science Research Unit Centre for Infectious Disease Research Lusaka Zambia; 5 School of Medicine Washington University St. Louis, MO United States; 6 School of Public Health Johns Hopkins University Baltimore, MD United States; 7 School of Medicine Georgetown University Washington, DC, DC United States; 8 School of Medicine University of Alabama Birmingham, AL United States; 9 School of Medicine University of California, Davis Sacramento, CA United States

**Keywords:** mHealth, mobile health, survey, incentives, HIV, Zambia, airtime, USSD, unstructured supplementary service data, HIV care, pilot study, mobile phone, public health service, mobile health, urban, rural, regression model, longitudinal, mobile, patient feedback

## Abstract

**Background:**

This pilot study evaluates the effectiveness of mobile talk-time incentives in maintaining participation in a longitudinal mobile health (mHealth) data collection program among people living with HIV in Lusaka, Zambia. While mHealth tools, such as mobile phone surveys, provide vital health feedback, optimal incentive strategies to ensure long-term engagement remain limited. This study explores how different incentive levels affect response rates in multiple survey rounds, providing insights into effective methods for encouraging ongoing participation, especially in the context of Zambia’s prepaid mobile system and multi-SIM usage, a common practice in sub-Saharan Africa.

**Objective:**

This study aimed to assess the response rate success across multiple invitations to participate in a care experience survey using a mobile phone short codes and unstructured supplementary service data (USSD) model among individuals in an HIV care setting in the Lusaka, Zambia.

**Methods:**

Participants were recruited from 2 study clinics–1 in a periurban setting and 1 in an urban setting. A total of 2 rounds of survey invitations were sent to study participants on a 3-month interval between November 1, 2018, and September 23, 2019. Overall, 3 incentive levels were randomly assigned by participant and survey round: (1) no incentive, (2) 2 Zambian Kwacha (ZMW; US $0.16), and (3) 5 ZMW (US $0.42). Survey response rates were analyzed using mixed-effects Poisson regression, adjusting for individual- and facility-level factors. Probability plots for survey completion were generated based on language, incentive level, and survey round. We projected the cost per additional response for different incentive levels.

**Results:**

A total of 1006 participants were enrolled, with 72.3% (727/1006) from the urban HIV care facility and 62.4% (628/1006) requesting the survey in English. We sent a total of 1992 survey invitations for both rounds. Overall, survey completion across both surveys was 32.1% (637/1992), with significantly different survey completion between the first (40.5%, 95% CI 37.4-43.6%) and second (23.7%, 95% CI 21.1-26.4) invitations. Implementing a 5 ZMW (US $0.42) incentive significantly increased the adjusted prevalence ratio (aPR) for survey completion compared with those that received no incentive (aPR 1.35, 95% CI 1.11-1.63). The cost per additional response was highest at 5 ZMW, equivalent to US $0.42 (72.8 ZMW [US $5.82] per 1% increase in response).

**Conclusions:**

We observed a sharp decline of almost 50% in survey completion success from the initial invitation to follow-up survey administered 3 months later. This substantial decrease suggests that longitudinal data collection potential for a care experience survey may be limited without additional sensitization and, potentially, added survey reminders. Implementing a moderate incentive increased response rates to our health care experience survey. Tailoring survey strategies to accommodate language preferences and providing moderate incentives can optimize response rates in Zambia.

**Trial Registration:**

Pan African Clinical Trial Registry PACTR202101847907585; https://pactr.samrc.ac.za/TrialDisplay.aspx?TrialID=14613

## Introduction

The increasing availability of mobile devices and mobile network coverage in sub-Saharan Africa presents a growing opportunity for mobile health (mHealth) interventions. By 2025, projections estimate that 50% of the sub-Saharan African population will own a mobile phone [1]. Zambia exemplifies this trend, with rapid mobile network expansion leading to an estimated 21.1 million mobile connections, reaching 91.4% of the population [2]. Mobile device usage in Zambia is shifting from voice calls to mobile internet, with users increasing from 9.1 million in 2019 to 12.5 million in 2023 [2,3]. In addition, the use of mobile money through local mobile network providers has risen rapidly, already 2.7 times higher in 2023 compared with 2021.

In much of sub-Saharan Africa, including Zambia, mobile network operators offer prepaid service plans. These plans allow users to purchase “talk-time,” providing finite minutes or data, as dedicated. With multiple competing mobile networks vying for market share, promotions and incentives for holding multiple phone numbers across the 3 main networks in Zambia are common. This can create challenges for mHealth engagement and response.

The rising level of accessibility to mobile devices allows for mHealth tools, such as mobile phone surveys, to capture important health-related feedback without substantially limiting participant eligibility. The use of the mHealth tools enables health care providers and health facility stakeholders to improve patient monitoring, enable mobile clinical decision support, and address contextual challenges in sub-Saharan Africa [4,5]. This technological advancement has the potential to greatly enhance health care outcomes by facilitating a better understanding of patient needs and improving service delivery in sub-Saharan Africa [6-8].

HIV care has now become chronic, with people making HIV care clinic visits every 3 months at the time of this research study in Zambia, providing an opportunity to regularly collect care experience measures. However, there is limited evidence in sub-Saharan Africa concerning the durability of an initial enrollment across multiple invitations and the role of incentives on survey response rates, especially in an era where mobile technology and mobile networks have become highly accessible. Previous work done in 4 countries in sub-Saharan Africa reports varying response rates to a general population SMS survey, with the highest response in Uganda (14.2%) and the lowest in Nigeria (0.3%) [9]. In 2016, a study in Kenya offering information on family planning methods, through SMS text messages, found that incentives varying between US $0.5 and US $1.0 were found to have no association with SMS survey response [10]. Another study in Zambia used SMS notifications as an option for linkage to care for individuals who are HIV positive [11]. Our study in Zambia is the first to examine the prospective outcomes of health-related surveys based on unstructured supplemental service data (USSD) at varying levels of talk-time incentives [12-14].

Using pilot data from a large stepped-wedge trial, we assess the pattern of responses across multiple rounds of invitation to participate in an mHealth model. Specifically, we investigated the association between talk-time incentive and rate of response to a brief, 4-question, USSD-based patient experience survey in Lusaka, Zambia. We collaborated with all the mobile network operators in Zambia to establish a unique USSD code (also called a short code) for the Person-Centered Public Health for HIV Treatment in Zambia (PCPH) study. We analyzed 3 levels of talk-time incentive, preferred language for survey administration, and clinic setting (periurban or urban) on the response rate of the USSD survey. Our goal was to evaluate the overall uptake, and response rate, to the survey after an HIV care visit in Lusaka, Zambia. Although this application was limited to HIV care, these findings may provide valuable insights for future initiatives involving mHealth in Zambia and sub-Saharan Africa.

## Methods

### Study Setting

This study was conducted as part of the pilot phase of the larger PCPH study, a cluster-randomized stepped-wedge trial to assess the impact of a patient-centeredness intervention on patient experiences of HIV care and outcomes, which ran from August 2018 to November 2021 in Lusaka Province (Pan African Clinical Trial Registry PACTR202101847907585) [15-17].

Data presented here were collected during the pilot phase, which was designed to develop and optimize a mobile phone–based survey for the main PCPH study, from November 1, 2018, to June 25, 2019. Pilot study clinics are run by the Zambian Ministry of Health (MoH) and receive various levels of technical support through the Centre for Infectious Disease Research in Zambia, an implementing partner of the MoH since 2011 and the primary implementer of the PCPH trial.

### Recruitment Procedure

Potential survey participants were identified by research assistants at 1 of 2 (1 in an urban setting, 1 in a periurban setting) pilot study HIV care facilities using convenience sampling; participants were selected following reception and triage procedures, which included routine data collection (eg, temperature, weight, and blood pressure). Eligibility for the study participation was limited to individuals who were enrolled as a recipient of care at the study facility, were aged 18 years or older, self-reported as literate in 1 of 4 survey languages, had a mobile phone during enrollment, and voluntarily consented to participate in the study. We confirmed that the mobile phone number reported by the potential participant belonged to the individual through a registration process using the unique PCPH short code, *744#, on the potential participant’s phone at enrollment. The registration process confirmed the ability to initiate and complete a USSD session, collected the language preference, and recorded the facility where the enrolment took place.

### Data Collection

#### Surveys

Survey participants were presented with 4 questions related to their experience at their most recent HIV care visit. The opening question was the same for all participants, the next 3 questions were drawn, using a systematic algorithm, from a bank of 9 questions (USSD survey question sets are shown in Multimedia Appendix 1). The survey questions were subject to a cognitive interviewing process to increase acceptability, understanding, and appropriateness before piloting. At enrollment, participants selected their preferred survey language from English, Bemba (spoken by 33.5% of the Zambian population), Nyanja (spoken by 14.8% of the Zambian population), or Tonga (spoken by 11.4% of the Zambian population). Although only 2% of the population have English as their first language, it is the most spoken second language [18].

#### Survey Administration

A total of 2 rounds of surveys were sent to those enrolled. The first round of survey invitations was sent through SMS text messages between November 1, 2018, and June 25, 2019, within 14 days of enrollment. The second round of invitations was sent 3 months after the first invitation, between February 1, 2019, and September 23, 2019. Survey spacing of 3 months was established through a review of median visit interval in the previous year at pilot study clinics. This second round was intended to capture the experience during the next routine HIV care visit, commonly scheduled quarterly (every 3 months). An incentive was systematically allocated to a participant once at registration for both survey rounds. The incentive allocation algorithm created a queue with values of 0 Zambian Kwacha (ZMW) or no incentives, 2 ZMW (US $0.16), and 5 ZMW (US $0.42), assigned in the order of enrollment. This queue was cycled by the incentive allocation algorithm, which allocated each incentive level before beginning at 0 at the end of each cycle. A currency exchange rate of 12.90 ZMW=US $1 was applicable and, at the time of the first round of the survey, 12.51 ZMW=US $1 was applicable [19]. In Zambia, talk-time is typically used to purchase bundles of minutes for voice calls, data, and sending and receiving SMS text messages. These bundles are valid for a day, 1 week, or 1 month. A 5 ZMW (US $0.42) talk-time can purchase a daily bundle with an average of 25 minutes for voice calls, 25 MB of data, and 127 SMS text messages. The survey invitation informed the survey participants of the incentive amount that they would receive upon survey completion. These incentive amounts were automatically sent to the participants through a third-party vendor immediately after the completion of all 5 survey questions. The money was sent as a talk-time top-up to study participants who all had a prepaid plan through their mobile network. Participants who did not start or complete the survey received a reminder through SMS text messages 24 hours after previous invitation and again 7 days after that invitation if the survey remained incomplete.

### Data Analysis

We assessed the association between survey completion and incentive amount for a USSD-based survey using a mixed-effects Poisson regression model, allowing random effects at both the individual and facility levels. A probability model with 95% CIs was illustrated for incentive level and language chosen by the participant. In addition, we projected the cost for additional response by incentive level based on 1000 invitations to the survey, by comparing the cost of additional response between 2 ZMW (US $0.16) and no incentives and between 5 ZMW (US $0.42) and no incentives. All analyses were completed using Stata BE (StataCorp LLC).

### Ethical Considerations

Ethical approval was granted by the University of Zambia Biomedical Research Ethics Committee (reference 008-03-19), the National Health Research Authority in Zambia, and the University of Alabama at Birmingham (300003282). The National Health Research Authority and the Zambian Ministry of Health provided authorization to conduct the research. The study participant data were deidentified, and a unique identifier was generated to identify participants’ responses across survey rounds. All the participants provided individual written informed consent before being enrolled in the study.

## Results

**Figure 1:**
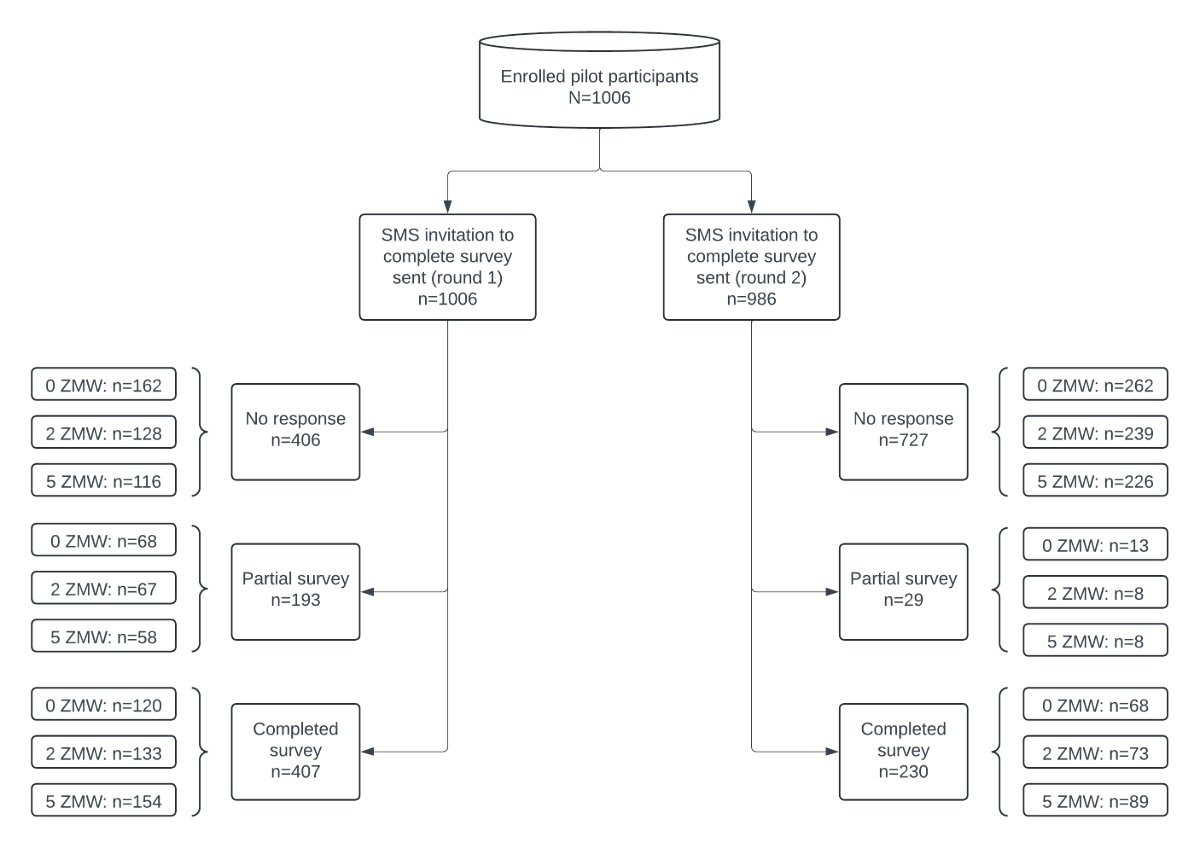
Participant response outcomes flow diagram by incentive level and survey round. Round 1 invitation to participate occurred within 14 days of enrollment in the study and round 2 occurred 3 months after round 1. ZMW: Zambian Kwacha.

A total of 1006 participants were enrolled, of which 986 (98%) received a second invitation to participate 3 months after the initial invitation, for a total of 1992 observations (Figure 1). At the beginning of the trial, the urban HIV care facility had a total of 7159 individuals receiving HIV care, while a periurban HIV care facility had a total of 3112 individuals receiving HIV care.

We found an overall response rate across both surveys to be 32% (637/1992) with significantly different response rates between the first (40.5%, 407/1006) and second round (23.3%, 230/986) surveys (*χ*^2^ *P*<.001; Tables S1 and S2 in Multimedia Appendix 1). The majority of those enrolled visited the urban-based HIV care facility (727/1006, 72.3%) and the survey was most often requested in English (628/1006, 62.4%; Table 1).

The second round of survey invitation had a significantly lower adjusted prevalence (adjusted prevalence ratio [aPR] 0.49, 95% CI 0.44-0.53) of response compared with the first round. Implementing a 5 ZMW (US $0.42) incentive significantly increased the prevalence ratio for survey completion compared with those that received no incentives (aPR 1.35, 95% CI 1.11-1.63; Table 2). Those enrolled at a visit to the urban study clinic had a significantly higher aPR at 1.26 (95% CI 1.03-1.52) compared with those at the periurban study clinic (Table 2).

**Figure 2:**
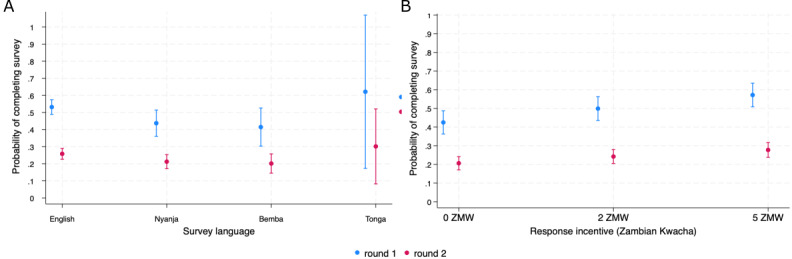
(A) Marginal probability of completing survey by language and survey round. (B) Marginal probability of completing the survey by incentive level and survey round. Round 1 occurred within 7 days of enrollment and round 2 occurred 3 months after the initial survey invitation of round 1. ZMW: Zambian Kwacha.

We observed a significant decrease in survey completion between survey 1 and the follow-up survey sent 3 months later. The probability of completing the survey was lowest for those who received no incentive in round 2 for which 20.6% completed the survey (95% CI 17.1-25.2; Figure 2; Table S3 in Multimedia Appendix 1).

We found a statistically significant association between language and setting for response to both survey invitations compared with just the first invitations. Individuals completing at least 1 survey in Bemba have a significantly lower adjusted prevalence (aPR 0.87, 95% CI 0.87-0.87) of completing both survey invitations compared with those completing the survey in English. In addition, individuals in urban settings have a significantly lower prevalence (aPR: 0.90, 95% CI 0.87-0.94 of responding to both survey invitations compared with individuals in a rural setting (Table 3).

Using observed response rates, we projected cost per additional response to be highest, at the 5 ZMW (US $0.42) incentive level, at 72.8 ZMW (US $5.82) per additional response as compared with the 0 ZMW incentive level considering 1000 invitations (Table 4).

**Table 1:** Participant characteristics by round of invitation (N_individuals_=1006).

Factors and their levels		Total invites (N=1992)	First round (n=1006)	Second round (n=986)
**Incentive, n (%)**				
	0 ZMW^a^	693 (34.8)	350 (34.8)	343 (34.8)
	2 ZMW (US $0.16)	648 (32.5)	328 (32.6)	320 (32.5)
	5 ZMW (US $0.42)	651(32.7)	328 (32.6)	323 (32.8)
**Response status, n (%)**				
	No response	1133 (56.9)	406 (40.4)	727 (73.7)
	Completed	637 (32.0)	407 (40.5)	230 (23.3)
	Partial	222 (11.1)	193 (19.2)	29 (2.9)
**Facility, n (%)**				
	Periurban	556 (27.9)	279 (27.7)	277 (28.1)
	Urban	1436 (72.1)	727 (72.3)	709 (71.9)
**Survey language, n (%)**				
	English	1240 (62.2)	628 (62.4)	612 (62.1)
	Nyanja	524 (26.3)	263 (26.1)	261 (26.5)
	Bemba	202 (10.1)	102 (10.1)	100 (10.1)
	Tonga	26 (1.3)	13 (1.3)	13 (1.3)

^a^ZMW: Zambian Kwacha.

**Table 2:** Crude and adjusted predictors of unstructured supplementary service data response. The model was adjusted for survey round, language, and setting; both unadjusted and adjusted models allow for random effects at individual and facility levels.

Factors and their levels			PR^a^ (95% CI)		aPR^b^, (95% CI)
**Survey round**					
	1	ref^c^		ref	
	2	0.48 (0.44-0.52)		0.49 (0.44-0.53)	
**Incentive amount**					
	0 ZMW^d^	ref		ref	
	2 ZMW (US $0.16)	1.17 (0.96-1.43)		1.17 (0.96-1.43)	
	5 ZMW (US $0.42)	1.35 (1.12-1.64)		1.35 (1.11-1.63)	
**Language**					
	English	ref		ref	
	Nyanja	0.77 (0.63-0.94)		0.75 (0.56-0.99)	
	Bemba	0.80 (0.60-1.06)		0.66 (0.43-1.03)	
	Tonga	0.98 (0.49-1.96)		1.08 (0.39-2.98)	
**Setting**					
	Periurban	ref		ref	
	Urban	1.28 (1.05-1.55)		1.26 (1.03-1.52)	

^a^PR: unadjusted prevalence ratio.

^b^aPR: adjusted prevalence ratio.

^c^ref: reference.

^d^ZMW: Zambian Kwacha.

**Table 3:** Predictors of responding to both survey invitations, compared with the first invite only (n=803).

Factors and their levels			aPR^a^ (95% CI)		*P* value
**Incentive amount**					
	0 ZMW^b^	ref^c^ (1.00)		—^d^	
	2 ZMW (US $0.16)	0.96 (0.77-1.21)		.75	
	5 ZMW (US $0.42)	1.03 (0.91-1.15)		.66	
**Language**					
	English	ref (1.00)		—	
	Nyanja	1.01 (0.92-1.11)		.86	
	Bemba	0.87 (0.87-0.87)		<.001	
	Tonga	0.97 (0.43-2.19)		.95	
**Setting**					
	Rural	ref (1.00)		—	
	Urban	0.90 (0.87-0.94)		<.001	

^a^aPR: adjusted prevalence ratio.

^b^ZMW: Zambian Kwacha.

^c^ref: reference.

^d^Not applicable.

**Table 4:** Cost for additional observations by incentive level based on 1000 invitations. Proportion increase compared with 0 ZMW^a^ incentive.

Incentive levels	Projected percent completing survey (N=1000), n (%)	Projected total cost (ZMW)	Projected percent completion increase	Projected price (ZMW) per percent increase given 1000 invitations
0 ZMW	341 (34.1)	0	0 (reference)	0 (reference)
2 ZMW (US $0.16)	403 (40.3)	258	6.2	41.6 ZMW (US $11.65) per 1% increase in response
5 ZMW (US $0.42)	464 (46.4)	750	12.3	72.8 ZMW (US $5.82) per 1% increase in response

^a^ZMW: Zambian Kwacha.

**Figure 3:**
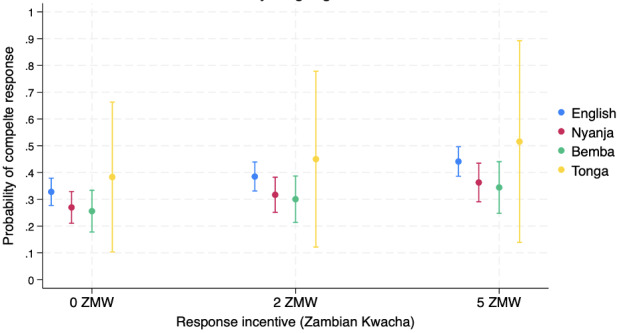
Marginal probability of responding to the survey by incentive level and language. ZMW: Zambian Kwacha.

Finally, we estimated the probability of survey completion by language and incentive level. English had a significantly higher completion rate than Bemba and Nyanja at all levels of the incentives with wide CIs around the Tonga point estimate (Figure 2). While not significant, survey participants who requested the survey in Tonga and were assigned 5 ZMW (US $0.42) had the highest estimated point probability of completing the survey, at 51.6% (95% CI 13.9-89.2), while those who spoke Bemba and received 0 ZMW had the lowest estimated point probability of completing the survey at 37.78% (95% CI 27.4-48.2; Figure 3; Table S4 in Multimedia Appendix 1). Incentive value was significantly associated with the response rate for those requesting the survey in English, while Bemba, Nyanja, and Tonga did not have a significant difference in survey completion probability by incentive (Figure 3).

## Discussion

### Principal Findings

We found a significant reduction in survey response rate across different languages and incentive levels 3 months after enrollment. Interestingly, the significant association between incentive level and survey response holds only for the first round of survey invitations, while there is no significant difference across incentive levels for the second round. Overall, the response rate across both surveys is highest (32.1%) among participants who received a 5 ZMW (US $0.42) incentive.

In Australia, longitudinal data collection through SMS text messages, with presurvey notifications, has been shown to be effective for monitoring postoperative pediatric pain, providing hope that our mHealth model may be an efficient mechanism to collect data longitudinally in our setting as well [20,21]. However, in our population of adults in HIV care, we found that subsequent invitations to participants were significantly less successful than the initial invitation. This restriction does not totally exclude the tool’s usage as a longitudinal data collection instrument. Some contributors to this response attrition may include (1) a high rate of phone number turnover, (2) phone discontinuance (phone sold, lost, stolen, etc), (3) no interest in the survey, (4) do not need incentive or incentive insufficient, and (5) no recent care experience to report in the event the participant has not had a return visit for HIV care. We suspect that phone number turnover is a chief contributor to the lower response rate to the second invitation given 1.1 mobile numbers per person (107.6 mobile network activations per 100 people) in Zambia in 2023 [2]. Unlike more expensive smartphones in many well-resourced settings, where mobile devices increasingly store vital information, such as credit cards and identifiable information, mobile devices in resource-limited settings tend to be more affordable [3]. In these settings, obtaining phone numbers often through prepaid schemes is inexpensive and readily accessible, and sometimes even provided for free through promotions by mobile network operators. Poor network quality and high cost of services are 2 other reasons leading to mobile customer churn in urban and periurban settings [22]. Although subsequent invitation response rates declined, it may remain a valuable method for collecting data at multiple time points, remotely (a vital aspect during health shocks like the COVID-19 pandemic) [23,24]. It is also possible that subsequent invitation response rates could be improved, despite phone number turnover, through routine updating of participant contact information.

While similar incentive-for-participation projects have been conducted in Zambia, such as those related to voluntary medical male circumcision, which used a 2 ZMW (US $0.16) incentive provided upon SMS survey completion, However, the response rate was not reported [25]. In our study, the provision of incentives was significantly associated with the response rate. There was a dose-response relationship observed between the incentive level and response rate; however, only the 5 ZMW (US $0.42) level was significantly different compared with no incentive. This is consistent with previous literature in Uganda, where incentivization for a general population survey on demographics, socioeconomic status, and technology was found to be significantly associated with response rate; however, incentive level appears to play a stronger role in response rate in our study than in the Uganda work [10]. In South Africa, a survey targeting randomly selected phone numbers from 4 mobile networks found that over 50% of participants opted for a prize raffle draw as an incentive compared with talk-time [26]. This is also the case in South Africa, where an SMS-based data collecting tool is used as part of a national surveillance system [27]. Future research may consider offering different types of incentives to participants who may not find talk-time a strong enough incentive to complete a survey.

Monetary incentives have been found to increase response rates for both web-based and paper-based surveys compared with no incentives in the United States [28]. It has also been shown that both pre- and postsurvey incentive provisions can yield similar participation [29]. In the United Kingdom, offering unconditional incentives before survey completion was found to be more effective than providing an incentive after survey completion [30]. Then, it is possible that the completion rates observed in this study could be further improved through unconditional survey incentives; however, further research is needed to confirm that the unconditional incentive completion results in the United Kingdom hold true in Zambia.

The pilot was conducted in an urban area at a clinic located within the capital city of Lusaka with full network coverage. In this setting, each participant would likely have more than 1 SIM card to take advantage of promotions offered on other networks. Residents in this community typically work within the city limits and would be able to receive survey participation prompt texts shortly after they are sent. On the other hand, our study clinic in the periurban area may have limited network coverage, being outside the city limits. Typically, families in these communities often work in more agricultural industries, which may lead to movement in and out of network coverage as they search for and conduct work in varying places. In this setting, participants likely switch between networks, and each with a different phone number, to use the network with available reception depending on location which may, in part, drive the poorer response rates we observe in the periurban clinic compared with the urban clinic.

We observed a significant difference in response rate across periurban and urban settings, which may be related to mobile device availability and literacy where individuals in urban settings may have increased exposure to mobile technology compared with those in periurban settings. Periurban settings, especially in resource-limited countries, have the greatest potential to benefit from mHealth efforts. However, sensitization to such technologies may need to be tailored to this population, which may have reduced familiarity with or access to mobile phone technology [12,31].

One strategy that can be used to increase response rates to these types of surveys is to offer incentives to participants who do not initially respond to the survey. There is evidence that this can work in a longitudinal survey conducted in the United States to people exposed to the 9/11 terrorist attacks [32]. This approach would result in a reduction in the cost of survey implementation. Gift cards have also shown evidence of a nonresponse conversation strategy in the United States [33].

In Zambia, English has been the medium of instruction in schools at all levels from primary, secondary, and tertiary education. However, it was only in 2013 when the new language policy came into effect that local languages started being used as a medium of instruction from grades 1 to 4 [34]. This explains why most survey participants chose to receive the survey in English. Literacy in English often means one can read in their native language.

The survey was offered in the 4 most common languages in Zambia with the lowest response rate for the first survey observed among the group requesting the survey in Nyanja (aPR: 0.75, 95% CI 0.56-0.99); however, the prevalence ratio for completing both surveys was higher, although not significantly so, for those requesting the survey in Nyanja (aPR: 1.01, 95% CI 0.43-2.19) compared with English. Although the majority opted to complete the survey in English (62.4%), a large proportion of responses among Nyanja and Bemba languages suggests that this is a useful tool to collect information from those who cannot, or prefer not to, read English in Zambia.

### Limitations

This study is subject to several limitations, the most substantial being that it is limited only to those with a mobile device. Although mobile technologies are becoming increasingly available in Zambia, there are still some that cannot yet be reached with this methodology. It is possible, because recruitment was based on convenience sampling, that the selection of participants was biased. However, given that we recruited 9.8% (1006/10,271) of the pilot clinic population and recruitment spanned almost 8 months (November 1, 2018, to June 25, 2019), we suspect the impact of potential selection bias to be minimal. Finally, we are limited in our model adjustment set as we did not collect participant demographic information, such as age, sex, and marital status, which may act as confounders and limit causal inference estimates. Our primary interest in the parent study was an ecological measure at the clinic level of patient experience and not the demographics of participants providing experience information.

### Conclusions

The study findings demonstrate the waning response rates over time, indicating a potential benefit in regular contact updates, as well as the differential effectiveness of incentives in improving survey response rates in the context of a mHealth platform for individuals in HIV care. In addition, they suggest that it is important to accommodate varying language preferences. The cost analysis also highlights the need for careful evaluation of the cost-effectiveness of different incentive levels, especially when baseline response rates without incentives are relatively high. Future research and larger-scale studies are necessary to assess the long-term effectiveness of longitudinal, incentive-based approaches in mHealth platforms, aiming to optimize health outcomes for individuals in HIV care and other health care populations.

## Appendix

Multimedia Appendix 1 Unstructured supplementary service data (USSD) survey question sets and supplemental tables.

## Abbreviations

aPR: adjusted prevalence ratio
mHealth: mobile health
MoH: Ministry of Health
PCPH: Person-Centered Public Health for HIV Treatment in Zambia
PR: prevalence ratio
SSA: sub-Saharan Africa
USSD: unstructured supplementary service data
Zambian Kwacha: ZMW

## Data Availability

The datasets generated during and/or analyzed during this study are available in the Data Dryad repository [35].
