# Supplementary material for: Assessing the Response Results of an mHealth-Based Patient Experience Survey Among People Receiving HIV Care in Lusaka, Zambia: Cohort Study
Source: J Med Internet Res. 2024 Sep 30;26:e54304. doi: 10.2196/54304 (PMC11474125; doi:10.2196/54304)
Supplement: Multimedia Appendix 1 [file jmir_v26i1e54304_app1.docx]

**Appendix: USSD Survey question sets**

Question set 1

1. Were you unhappy with the care you received?
2. Did you see health care workers behave rudely?
3. Did the facility find your file at your visit?",
4. Did you spend more than four hours at the clinic?

Question set 2

1. Were you unhappy with the care you received?
2. Would you encourage your friend to attend this facility?
3. Did you receive one month or less of medicine?
4. Will it be difficult for you to attend your next clinic appointment?

Question set 3

1. Were you unhappy with the care you received?
2. Would you encourage your friend to attend this facility?
3. Did your care provider listen to what you had to say?
4. Did you see health care workers behave rudely?

**Table S1. Crude and adjusted regression results for survey response round one.**

| *Covariate* | *Level* | Unadjusted | | Adjusted | |
| --- | --- | --- | --- | --- | --- |
|  |  | *PR* | *95% CI* | *aPR* | *95% CI* |
| Incentive Amount | 0 ZMW | ref | ref | ref | ref |
|  | 2 ZMW | 1.20 | (1.17, 1.23) | 1.20 | (1.00, 1.43) |
|  | 5 ZMW | 1.34 | (1.27, 1.41) | 1.33 | (1.12, 1.58) |
| Language | English | ref | ref | ref | ref |
|  | Nyanja | 0.85 | (0.81, 0.90) | 0.87 | (0.73, 1.04) |
|  | Bemba | 0.87 | (0.78, 0.99) | 0.85 | (0.66, 1.10) |
|  | Tonga | 1.14 | (0.77, 1.69) | 1.22 | (0.63, 2.38) |
| Setting | Peri-urban | ref | ref | ref | ref |
|  | Urban | 1.29 | (1.29, 1.29) | 1.29 | (1.08, 1.54) |

Note: PR – prevalence ratio, aPR – adjusted prevalence ratio, adjustment set – language and setting, random effects allowed at facility level for all models.

**Table S2. Crude and adjusted regression results for survey response round two.**

| *Covariate* | *Level* | Unadjusted | | Adjusted | |
| --- | --- | --- | --- | --- | --- |
|  |  | *PR* | *95% CI* | *aPR* | *95% CI* |
| Incentive Amount | 0 ZMW | ref | ref | ref | ref |
|  | 2 ZMW | 1.14 | (0.85, 1.51) | 1.14 | (0.85, 1.53) |
|  | 5 ZMW | 1.37 | (1.21, 1.55) | 1.37 | (1.04, 1.81) |
| Language | English | ref | ref | ref | ref |
|  | Nyanja | 0.73 | (0.69, 0.77) | 0.75 | (0.56, 0.99) |
|  | Bemba | 0.69 | (0.63, 0.76) | 0.66 | (0.43, 1.03) |
|  | Tonga | 0.93 | (0.42, 2.07) | 1.08 | (0.39, 2.98) |
| Setting | Peri-urban | ref | ref | ref | ref |
|  | Urban | 1.21 | (1.21, 1.21) | 1.20 | (0.92, 1.57) |

Note: PR – prevalence ratio, aPR – adjusted prevalence ratio, adjustment set – language and setting, random effects allowed at facility level for all models.

**Table S3. Marginal probability estimates of survey completion with interaction terms for survey round and incentive level and survey round and language.**

| Round | *Term* | *Probability* | *95% CI* | *p-value* |
| --- | --- | --- | --- | --- |
| Round 1 | English | 0.5316 | (0.4887, 0.5746) | <0.001 |
|  | Nyanja | 0.4373 | (0.3604, 0.5143) | <0.001 |
|  | Bemba | 0.4147 | (0.3032, 0.5262) | <0.001 |
|  | Tonga | 0.6213 | (0.1728, 1.0697) | 0.007 |
|  | 0 ZMW | 0.4250 | (0.3629, 0.4871) | <0.001 |
|  | 2 ZMW | 0.4992 | (0.4354, 0.5629) | <0.001 |
|  | 5 ZMW | 0.5719 | (0.5088, 0.635) | <0.001 |
| Round 2 | English | 0.2580 | (0.2263, 0.2898) | <0.001 |
|  | Nyanja | 0.2123 | (0.1713, 0.2533) | <0.001 |
|  | Bemba | 0.2013 | (0.1450, 0.2576) | <0.001 |
|  | Tonga | 0.3015 | (0.0824, 0.5207) | 0.007 |
|  | 0 ZMW | 0.2063 | (0.171, 0.2415) | <0.001 |
|  | 2 ZMW | 0.2423 | (0.2049, 0.2797) | <0.001 |
|  | 5 ZMW | 0.2776 | (0.238, 0.3172) | <0.001 |

Note: CI - confidence interval.

**Table S4. Marginal probability estimates of survey completion with interaction terms for language and incentive level.**

| *Factor* | *Term* | *Probability* | *95% CI* | *p-value* |
| --- | --- | --- | --- | --- |
| Bemba | 0 ZMW | 0.2557 | (0.1777, 0.3338) | <0.001 |
|  | 2 ZMW | 0.3004 | (0.214, 0.3867) | <0.001 |
|  | 5 ZMW | 0.3441 | (0.2476, 0.4407) | <0.001 |
| English | 0 ZMW | 0.3279 | (0.2768, 0.3789) | <0.001 |
|  | 2 ZMW | 0.3851 | (0.331, 0.4392) | <0.001 |
|  | 5 ZMW | 0.4412 | (0.3862, 0.4962) | <0.001 |
| Nyanja | 0 ZMW | 0.2697 | (0.2105, 0.3289) | <0.001 |
|  | 2 ZMW | 0.3168 | (0.2513, 0.3823) | <0.001 |
|  | 5 ZMW | 0.3629 | (0.2908, 0.435) | <0.001 |
| Tonga | 0 ZMW | 0.3831 | (0.1032, 0.6631) | 0.007 |
|  | 2 ZMW | 0.4500 | (0.1216, 0.7784) | 0.007 |
|  | 5 ZMW | 0.5156 | (0.1389, 0.8922) | 0.007 |

Note: CI - confidence interval.
